# Curative Radical Jejunectomy for a Refractory Upper Gastrointestinal Bleed in a HeartMate 3 Left Ventricular Assist Device (LVAD) Patient: A Case Report

**DOI:** 10.7759/cureus.90287

**Published:** 2025-08-17

**Authors:** Rangish Yuvaraj, Havish S Kantheti, Aasim Afzal, David Rawitscher, Salman Gohar

**Affiliations:** 1 Internal Medicine, Baylor Scott & White Health, Fort Worth, USA; 2 Cardiology, The Heart Hospital Plano, Baylor Scott & White Heart and Vascular Hospital, Plano, USA; 3 Advanced Heart Failure and Cardiac Transplantation, Baylor Scott & White Health, Fort Worth, USA

**Keywords:** gi bleed, heart mate 3, heart transplant, jejunectomy, left ventricular assist device (lvad)

## Abstract

Gastrointestinal bleeding (GIB) is a known complication in patients with left ventricular assist devices (LVADs), and can be difficult to manage when bleeding is recurrent or unresponsive to standard therapies. We describe the case of a 63-year-old woman with a HeartMate 3 LVAD who presented with repeated episodes of GIB. Extensive evaluation revealed jejunal angiodysplasia as the source. Despite multiple endoscopic treatments and temporary discontinuation of anticoagulation, the bleeding persisted. Ultimately, the patient underwent exploratory laparotomy with resection of approximately 100 cm of proximal jejunum, which successfully resolved the bleeding. This case demonstrates that surgical intervention can be an effective option for refractory GIB due to angiodysplasia in LVAD patients, and underscores the importance of a multidisciplinary approach when balancing bleeding and thrombotic risks.

## Introduction

Left ventricular assist device (LVAD) therapy serves as a bridge to transplantation or as destination therapy, significantly improving survival in patients with end-stage heart failure. The HeartMate 3, a magnetically levitated, centrifugal-flow LVAD, has demonstrated reduced thrombosis and stroke rates compared to previous generations, yet gastrointestinal bleeding (GIB) remains a prevalent complication. In the MOMENTUM 3 trial, the cumulative incidence of GIB reached 24.5% at five years [1].

The etiology of GIB in LVAD patients is multifactorial. Contributing factors include acquired von Willebrand syndrome due to shear stress-induced proteolysis of von Willebrand factor (vWF), formation of arteriovenous malformations (AVMs), diminished arterial pulsatility, and chronic anticoagulation [2]. Angiodysplasia, a form of AVM characterized by dilated, fragile blood vessels in the gastrointestinal mucosa and submucosa, is particularly implicated. Continuous-flow devices have been shown to promote mucosal hypoperfusion and vascular remodeling, predisposing patients to these lesions [3]. Although GIB may originate throughout the gastrointestinal tract, approximately 33% of lesions are upper GI, 25% lower, and nearly half present without an identifiable source, despite extensive workup [4].

Management strategies include anticoagulation adjustment and pharmacologic agents such as danazol, thalidomide, octreotide, desmopressin, and bevacizumab to reduce AVM burden [4]. Endoscopic interventions provide temporary control but are often followed by recurrent bleeding. In rare and refractory cases, surgical resection of the bleeding source may be required. However, data on the efficacy and long-term outcomes of such interventions, especially in HeartMate 3 patients, remain limited.

Here, we present a case of a patient with a HeartMate 3 LVAD who experienced recurrent GIB due to extensive small bowel angiodysplasia, ultimately requiring proximal jejunectomy, which resulted in complete resolution of bleeding.

## Case presentation

A 63-year-old female with nonischemic cardiomyopathy, requiring an HM3 LVAD implant approximately three years ago, had her clinical course in 2022 complicated by a multi-drug resistant (MDR) *Pseudomonas aeruginosa* driveline infection and bacteremia. She presented for repeat admission with lightheadedness, dizziness, fatigue, exertional dyspnea, and melena.

She initially began having these symptoms approximately six months after LVAD implantation and underwent enteroscopy three times, which revealed no upper GI sources of bleeding, no AVMs, and a normal jejunum (Table 1). On admission, her INR was 4.0; thus, her warfarin 5 mg and acetylsalicylic acid (ASA) were held. She was discharged after bleeding resolved following 4 units of packed red blood cells (pRBCs), with resumption of her 5 mg warfarin dose and an INR goal of 2.0.

**Table 1:** Chronologic Timeline of Endoscopic Interventions and Findings for Recurrent Gastrointestinal Bleeding This table outlines the patient's diagnostic workup for recurrent gastrointestinal bleeding. AVM, arteriovenous malformation; RBC, red blood cell

Date	Diagnostic Intervention and Findings
12/29/22	Small bowel enteroscopy revealed no upper GI sources, no AVMs, jejunum was normal.
1/17/23	Small bowel enteroscopy was unremarkable, with no AVMs, no stigmata of bleeding. Plans for capsule endoscopy and tagged RBC scan if positive.
3/9/23	Small bowel enteroscopy with no evidence of bleeding, no AVMs, normal duodenum, and jejunum.
8/12/24	Small bowel enteroscopy showed no evidence of pathology in the proximal jejunum, approximately 40 cm distal to the ligament of Treitz. No AVMs were identified.
10/26/24	Small bowel enteroscopy with normal pathology.
10/27/24	Pill cam study with small bowel bleeding in the mid-jejunum. Referred for double balloon enteroscopy.
10/27/24	Small bowel enteroscopy, which demonstrated no bleeding.
11/13/24	Small bowel enteroscopy found mid-ileum small angioectasia with bleeding, 3 clips placed, and the area tattooed with India ink. The rest of the jejunum is normal.
1/5/25	Small bowel enteroscopy with patchy erythematous mucosa in the gastric body. No significant pathology in the proximal jejunum, another area marked with an India ink tattoo.
1/15/25	Small bowel enteroscopy showed mid-ileum angioectasia with bleeding in close proximity to previous clip placement. Another hemostatic clip was placed, and 2 mL of epinephrine solution was injected at the site for hemostasis. The rest of the jejunum is normal.
1/24/25	Exploratory laparotomy with small bowel resection; approximately 100 cm of proximal jejunum was removed. A wound vac was applied. The jejunum was speckled with tiny luminal AVMs, while the ileum was completely normal all the way to the ileocecal valve. Diffuse coagulopathy was noted from all surfaces throughout the operation.

Unfortunately, she continued to have melena, requiring another admission less than a month later. She presented with an initial hemoglobin of 5.7 g/dL, requiring 5 units of pRBCs during her stay. This time, she was discharged home on a reduced warfarin dosage of 2 mg, with outpatient follow-up in a week.

Three months later, she again presented with a similar scenario, with an initial hemoglobin of 5.2 g/dL and an INR of 1.5. A subsequent enteroscopy found no stigmata of bleeding. A capsule endoscopy identified a small bowel bleed in the mid-jejunum, and three hemostasis clips were placed. Warfarin was discontinued, she was transitioned to Eliquis 2.5 mg, and discharged home with a three-week outpatient follow-up.

She remained stable for five months, then began having melena again. She was referred for a double-balloon enteroscopy to a center with a higher level of care, due to suspected bleeding in the distal jejunum and proximal terminal ileum, which could not be explored after her sixth conventional enteroscopy, with plans to hold Eliquis for a month. Unfortunately, she experienced another bleeding episode before restarting Eliquis. On her sixth admission, enteroscopy revealed a mid-ileum angioectasia with bleeding near the previous clip.

**Figure 1:**
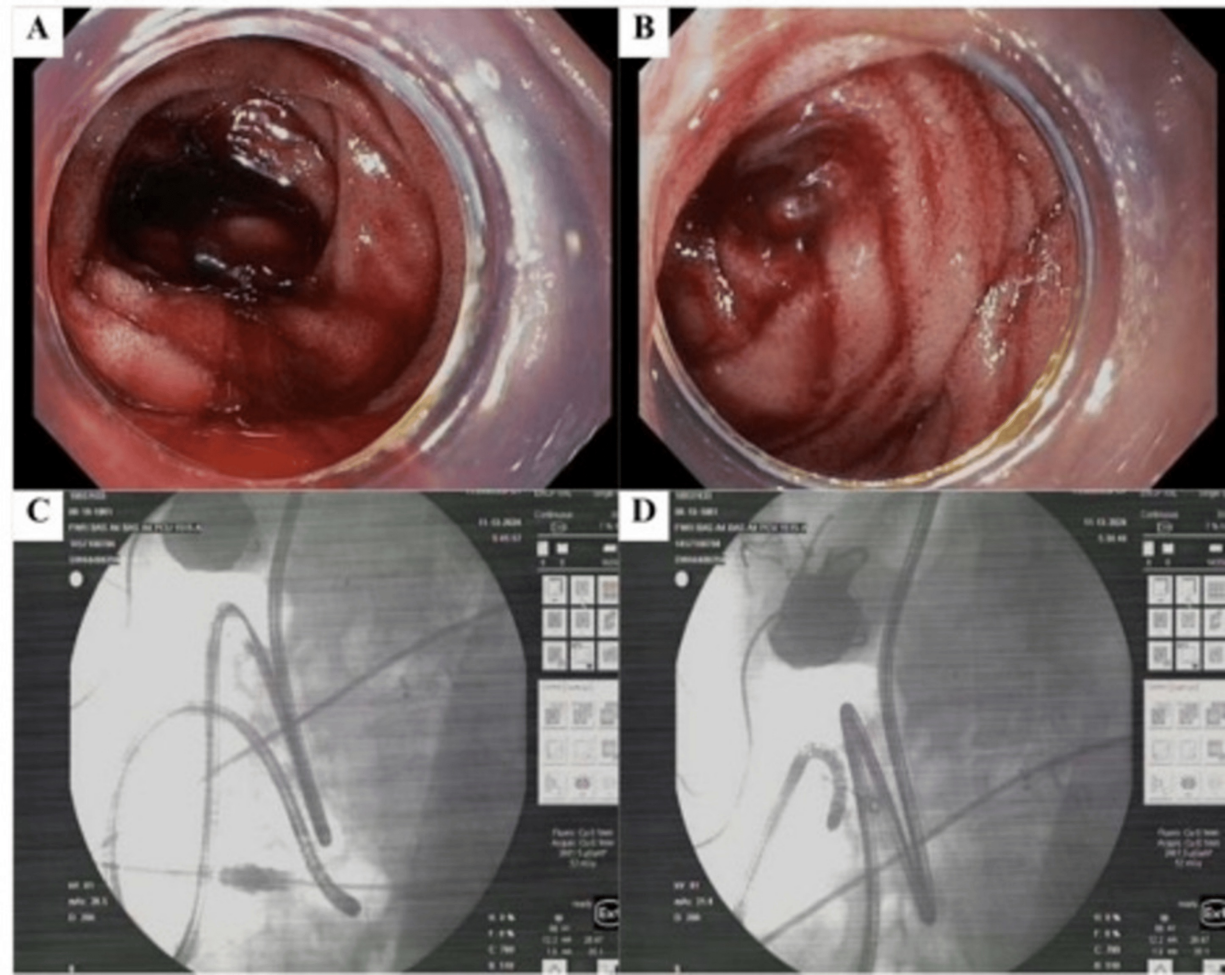
Double Balloon Enteroscopy Revealing Angioectasia and Luminal AVMs (A) Mid-ileum angioectasia with bleeding; (B) Jejunectomy site: speckled luminal AVMs; (C) Double-balloon enteroscopy view of the mid-ileum, highlighting clustered angioectasias; (D) Double-balloon enteroscopy image at the jejunectomy site, showing diffuse luminal AVMs AVMs, arteriovenous malformations

She was discharged with no anticoagulation, and it was decided that an exploratory laparotomy was prudent to visualize the adjacent area previously tattooed in her jejunum on 11/13/24, with the possibility of a small bowel resection for definitive management. The resection showed that her jejunum was extensively speckled with tiny luminal AVMs, while the ileum was normal all the way to the ileocecal valve (Figure 1). She had diffuse oozing from mucosal surfaces upon visual inspection. She underwent a proximal resection of 100 cm of her jejunum without complications, which resolved further bleeding episodes (Figure 1). 

## Discussion

GIB is a well-recognized complication in LVAD patients, affecting morbidity and quality of life. The continuous flow of modern LVADs contributes to altered shear stress, leading to acquired von Willebrand syndrome and increased susceptibility to bleeding. Studies have demonstrated that the degradation of vWF in LVAD patients promotes angiogenesis, predisposing them to the development of AVMs and subsequent bleeding [5]. A direct correlation between the reduction of revolutions per minute (RPM), significantly reducing vWF degradation, was demonstrated [6]. Additionally, the low pulsatility state induced by an LVAD contributes to AVM formation, exacerbating the risk of recurrent bleeding [7].

The management of LVAD-associated GIB remains challenging due to the competing risks of bleeding and thromboembolism. While pharmacologic therapies such as octreotide, thalidomide, and bevacizumab have been explored, their effectiveness remains variable [8]. Endoscopic interventions, including argon plasma coagulation and clipping, may provide temporary relief, but the recurrence rate remains high, necessitating repeated procedures [9]. In cases of refractory bleeding, surgical resection may be the only curative option.

This case highlights the complexity of balancing anticoagulation management and bleeding risk in LVAD patients. Despite the initial decision to withhold anticoagulation due to the patient’s history of intracranial hemorrhage, careful resumption of therapy was achieved without subsequent thrombotic events. The decision to proceed with jejunectomy was made after repeated failures of medical and endoscopic therapy, emphasizing the role of surgical intervention in select cases of transfusion-dependent GIB. A multidisciplinary approach, involving heart failure specialists, gastroenterologists, and surgical teams, is essential in guiding management decisions and optimizing patient outcomes [10].

Further research is needed to establish standardized treatment protocols for LVAD-associated GIB, particularly in cases refractory to pharmacologic and endoscopic interventions. Understanding the long-term outcomes of surgical interventions, such as jejunectomy, will be critical in refining management strategies for this challenging patient population.

## Conclusions

Recurrent GIB remains a major complication of LVAD therapy, often necessitating a multimodal approach. In cases of transfusion-dependent, refractory AVM-related bleeding, surgical resection may serve as a definitive treatment option after the failure of medical and endoscopic therapies. This case illustrates the potential role of jejunectomy in managing persistent GIB in a HeartMate 3 patient, with complete resolution of bleeding postoperatively. However, given the single-patient nature of this report and limited follow-up duration, generalizability remains limited. This case underscores the need for individualized decision-making, supported by a multidisciplinary team - including cardiology, gastroenterology, and surgery - when weighing bleeding risk against thrombotic complications. Further research is warranted to better define outcomes and long-term risks of surgical intervention in this complex patient population.
